# Synthesis and In Vitro Evaluation of Novel Nortropane Derivatives as Potential Radiotracers for Muscarinic M_2_ Receptors

**DOI:** 10.1155/2011/709416

**Published:** 2011-06-13

**Authors:** Remco J. J. Knol, Jan C. van den Bos, Anton G. M. Janssen, Kora de Bruin, Berthe L. F. van Eck-Smit, Jan Booij

**Affiliations:** ^1^Department of Nuclear Medicine, Medical Center Alkmaar, Wilhelminalaan 12, 1815 JD Alkmaar, The Netherlands; ^2^Department of Organic Chemistry, Eindhoven University of Technology, Den Dolech 2, 5600 MB Eindhoven, The Netherlands; ^3^GE Healthcare, Cygne Center, De Rondom 8, 5612 AP Eindhoven, The Netherlands; ^4^Department of Nuclear Medicine, Academic Medical Center, University of Amsterdam, Meibergdreef 9, 1105 AZ Amsterdam, The Netherlands

## Abstract

Disturbances of the cerebral cholinergic neurotransmitter system are present in neurodegenerative disorders. SPECT or PET imaging, using radiotracers that selectively target muscarinic receptor subtypes, may be of value for in vivo evaluation of such conditions. 6**β**-acetoxynortropane, a potent muscarinic M_2_ receptor agonist, has previously demonstrated nanomolar affinity and high selectivity for this receptor. Based on this compound we synthesized four nortropane derivatives that are potentially suitable for SPECT imaging of the M_2_ receptor. 6**β**-acetoxynortropane and the novel derivatives were tested in vitro for affinity to the muscarinic M_1−3_ receptors. The original 6**β**-acetoxynortropane displayed high affinity (*K*_*i*_ = 70–90 nM) to M_2_ receptors and showed good selectivity ratios to the M_1_ (65-fold ratio) and the M_3_ (70-fold ratio) receptors. All new derivatives showed reduced affinity to the M_2_ subtype and loss of subtype selectivity. It is therefore concluded that the newly synthesized derivatives are not suitable for human SPECT imaging of M_2_ receptors.

## 1. Introduction

Central cholinergic disturbances are present in many neuropsychiatric and neurodegenerative diseases. In various forms of dementia, such as Alzheimer's dementia (AD) or Lewy body dementia, cholinergic deficits in the brain [1, 2] are associated with cognitive decline [3–5] and are thought to precede clinical symptoms. 

The majority of the cholinergic deficits in these diseases arise from degenerative events in basal forebrain regions such as the nucleus basalis of Meynert [6, 7], which provides the cholinergic input of the cerebral cortex. In degenerative diseases such as AD, disruption of basal cholinergic forebrain projections leads to a presynaptic cholinergic defect in cortical brain areas [1, 8]. Being part of a family of five muscarinic receptor subtypes, the muscarinic M_2_ receptor is located predominantly presynaptically [9] and is consequently a potential target for the evaluation of the integrity of the cholinergic neurotransmitter system by molecular imaging.

In vivo assessment of the central cholinergic system in patients suffering from dementia by means of positron emission tomography (PET) or single photon emission computed tomography (SPECT) may be of value for early diagnosis or monitoring of such diseases, to predict response to cholinergic therapies (such as acetylcholinesterase inhibitors) or to evaluate effects of experimental drugs. Molecular imaging of the cholinergic system of the brain requires radiotracers that ideally selectively target specific neuroreceptors of this neurotransmitter system, such as the muscarinic M_2_ receptor. 

Many attempts have been made to develop muscarinic receptor subtype selective radiotracers [10–12]. Currently, amongst the most promising of these potential tracers is [^18^F]FP-TZTP [10, 13, 14], which has selectivity for the muscarinic M_2_ receptor [15] and has been applied successfully in several human PET studies [16–18]. 

Iodine-123 has favorable properties for SPECT imaging of neuroreceptors [19–23]. The abundant 159 keV *γ* photons of ^123^I (83% abundancy) are suitable for high-resolution brain SPECT imaging using LEHR (Low Energy High Resolution) or fanbeam collimators. Furthermore, unlike ^18^F-labeled radiopharmaceuticals (*T*_1/2_ = 109.8 min), the half-life of 13.2 hours permits transportation over long distances of centrally produced ^123^I-labeled radiopharmaceuticals, for instance, to the majority of European nuclear medicine centers from a single manufacturing site. Moreover, suitable methods have been developed for convenient radiosynthesis of radiopharmaceuticals labeled with ^123^I selectively at one appropriate position with high radiochemical yields, for instance, by oxidative iododestannylation [24]. However, radioiodinated SPECT analogues of TZTP so far have demonstrated disappointing results in vivo [25]. Radioiodinated Z-IQNP is another compound that has recently been evaluated for imaging of the muscarinic M_2_ receptor [12, 26], although the muscarinic subtype selectivity of this compound for the M_2_ receptor subtype is limited. 

Recently, 6*β*-acetoxynortropane, a tropane alkaloid, was reported to be a potent and highly selective agonist for muscarinic M_2_ receptors [27], and radiolabeled derivatives of this compound may thus be of value for in vivo imaging of these receptors. Moreover, as compared with the mentioned TZTP analog these nortropane analogs have favorable physicochemical properties. Notably, the lipophilicity of iodinated nortropane compounds remains in the optimal range 1–3 of log*D*  (pH 7.4). The log*D* (pH 7.4) of iodinated TZTP compounds, on the other hand, is too high (i.e., >4), likely resulting in a high degree of nonspecific binding in the brain. In order to obtain good in vivo stability, the iodine label should be bound to an sp2-carbon, where iodoallyl- and iodophenyl compounds are the most suitable candidates. Of these, iodophenyl compounds are synthetically the most accessible and more stable. 

Based on 6*β*-acetoxynortropane, we synthesized four derivatives as potential radiotracers for use in SPECT imaging. The four synthesized nortropane derivatives were tested for affinity to cloned human muscarinic M_1_–M_3_ receptor subtypes on membrane fractions of Chinese hamster ovary (CHO) cells by in vitro competitive binding assays.

## 2. Material and Methods

**Figure 1:**
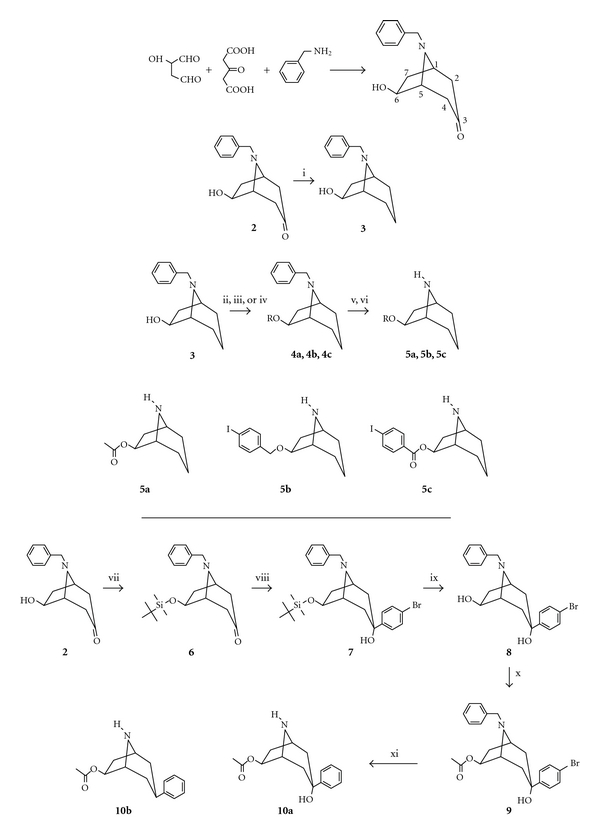
Reagents and conditions: (i) NH_2_NH_2_, NaOH; (ii) Ac_2_O, pyridine; (iii) 4-Iodobenzyl bromide, DMF; (iv) 4-Iodobenzoyl chloride, DMAP, Et_3_N, CH_2_Cl_2_; (v) *α*-chloroethyl chloroformate, Toluene; (vi) MeOH; (vii) TBDMSCl, DMAP, Et_3_N, DMF; (viii) Mg, 1,4-dibromobenzene, THF; (ix) HCl (2 M), THF, EtOH (1/1/1); (x) Ac_2_O, pyridine; (xi) H_2_, Pd/C. Variants **4a–c**: R (**a**) CH_3_CO, (**b**) *p*-IPhCH_2_, and (**c**) *p*-IPhCO.

Two nortropane analogues with an iodine containing moiety on the 6*β*-position have been synthesized. The tropane skeleton was formed in a single-step multicomponent reaction in analogy to the classical Robinson tropinone synthesis [28, 29], as displayed in Figure 1. The resulting tropinone was reduced under Wolff-Kishner conditions to give 6-hydroxy-N-benzylnortropane (**3**). Alkylation or acylation of the hydroxyl function of **3** resulted in **4a–c, **which were debenzylated in two steps [29, 30] to provide the previously described 6*β*-acetoxynortropane (**5a**), and its iodinated analogues, the 6*β*-4′-iodobenzyl ether (**5b**) or the 6*β*-4′-iodobenzoate ester (**5c**) of 6*β*-nortropinol, respectively.

Accordingly, a bromophenyl ring was introduced at the 3*β*-position. First, the 6-hydroxyfunction of tropinone (**2**) was protected as tert-butyldimethylsilyl ether. With a Grignard reaction 4-bromophenyl was introduced at the C3 of the tropane. According to the signal of the C6*α*-H in the proton NMR spectrum, only the isomer with the 4-bromophenyl in the equatorial position had been formed. Desilylation followed by acetylation yielded 3*β*-(4-bromophenyl)-6*β*-acetoxy-N-benzyl nortropane (**9**).

The benzyl group was removed by hydrogenation, but simultaneously also the bromo substituent was removed to result in 3*α*-hydroxy-3*β*-phenyl-6*β*-acetoxy-nortropane (**10b**). In one occasion also the 3*β*-phenyl-6*β*-acetoxy-nortropane (**10a**) was isolated, presumably due to the presence of a small amount of acid.

Relative binding affinity and selectivity ratios of the various nortropane derivatives for the M_1_–M_3_ were determined by competitive binding assays against [^3^H]N-methylscopolamine ([^3^H]NMS, Perkin Elmer, Waltham, USA; specific activity 78 Ci (2886 GBq)/mmol). Assays were performed on membrane suspensions from CHO cells expressing either the recombinant human muscarinic M_1_, M_2,_ or the M_3_ receptor subtype (Perkin Elmer, Waltham, USA) [25]. 

In the competitive binding assays, incubation buffer contained 50 mM TRIS-HCl, 10 mM MgCl_2_, and 1 mM EDTA (pH 7.4 at 4°C). The assays were incubated during 60 min (M_1_ or M_2_ receptor subtypes) or 120 min (M_3_ receptor subtypes) at 27°C. Nonspecific binding was determined using atropine as a competitor in a concentration of 1 *μ*M.

In the first series of competitive binding assays, the 6*β*-4′-iodobenzyl ether (**5b**) and the 6*β*-4′-iodobenzoate ester (**5c**) of 6*β*-nortropinol, as well as the lead compound 6*β*-acetoxynortropane (**5a**), were tested. Protein concentrations for undiluted receptor subtype suspensions were 1.2 mg/mL (M_1_), 4.3 mg/mL (M_2_), and 2.0 mg/mL (M_3_). Aliquots (*n* = 3) of diluted membranes (factor 1 : 100) containing the M_1_–M_3_ receptor subtypes were incubated in a total volume of 540 *μ*L containing 500 *μ*L diluted membranes, 20 *μ*L [^3^H]NMS, and 20 *μ*L of the nortropanes in increasing concentrations. The [^3^H]NMS was used in a final concentration of 0.2 nM for the M_1_ and M_2_ assays and 0.09 nM for the M_3_ assays. The equilibrium dissociation constants in nM of [^3^H]NMS for the three receptor subtypes, provided by the manufacturer, were 0.15 (M_1_), 0.19 (M_2_), and 0.08 (M_3_). Final competitor concentrations ranged from 1.0 · 10^−10^ M to 1.0 · 10^−4^ M. After incubation, the reaction was rapidly terminated by vacuum filtration over GF/C glass fiber filters, presoaked in 0.3% polyethylenimine (Sigma-Aldrich, Munich, Germany), and washed 5 times with 1 mL of ice-cold buffer. Filters were placed in vials with 10 mL of scintillation fluid (Ultima Gold, Perkin Elmer, Waltham, USA) and counted in a liquid scintillation counter (Tri-Carb 2900 TR Liquid Scintillation Analyzer, Packard. Software version: 3100). 

In the second series of competitive binding assays, derivative **10a** and **10b** and the lead compound 6*β*-acetoxynortropane (**5a**) were tested. In this series, the protein concentrations for undiluted receptor subtype suspensions were 0.6 mg/mL (M_1_), 7.5 mg/mL (M_2_), and 1.5 mg/mL (M_3_). Aliquots (*n* = 4) of diluted membranes (factor 1 : 30) containing the M_1_, M_2_, or M_3_ receptor subtype were incubated on a microplate in volumes of 190 *μ*L containing 150 *μ*L diluted membranes, 20 *μ*L [^3^H]NMS, and 20 *μ*L of the nortropanes in increasing concentrations. In these assays, the [^3^H]NMS was used in a final concentration of 0.13 nM for the M_1_ and M_2_ assays and 0.065 nM for the M_3_ assays. The *K*_*d*_ in nM of [^3^H]NMS in these experiments were as stated above. Final competitor concentrations ranged from 1.0 · 10^−10^ M to 1.0 · 10^−5^ M. After incubation, the assays were filtrated over UniFilter 96 GF/C filter plates, presoaked in 0.3% polyethylenimine (Sigma-Aldrich, Munich, Germany), and washed 9 times with 200 *μ*L of ice-cold buffer. 30 *μ*L of scintillation fluid (MicroScint, Perkin Elmer, Waltham, USA) was added, and the filter plates were counted in a liquid scintillation counter (TopCount 5.0 Liquid Scintillation Analyzer, Perkin Elmer, Waltham, USA). For each competitor, the inhibition constant (*K*_*i*_) was calculated from the EC_50_ for the muscarinic M_1_, M_2_, and M_3_ subtypes with nonlinear regression curve fitting using Graphpad Prism (version 3.02), relative to the *K*_*d*_ of [^3^H]NMS as provided by the manufacturer.

## 3. Results

**Figure 2:**
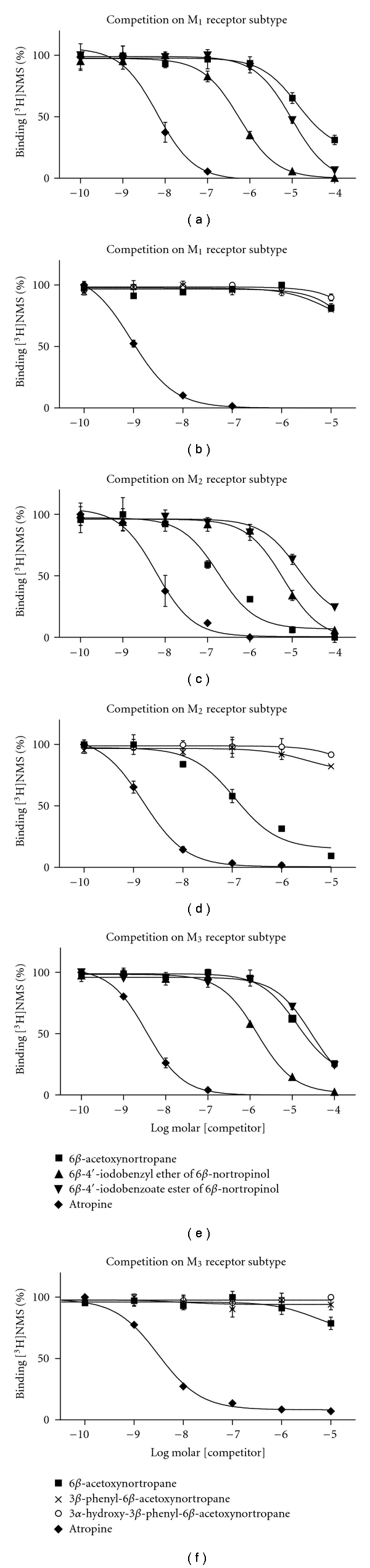
Competition curves of 6*β*-acetoxynortropane (**5a**, *K*_*i*_ for M_2_ in two separate experiments 88.1 nM and 71.6 nM, resp.) and the 6*β*-4′-iodobenzyl ether (**5b**, *K*_*i*_ for M_2_ 3.0 *μ*M) and 6*β*-4′-iodobenzoate ester (**5c**, *K*_*i*_ for M_2_ 6.8 *μ*M) of 6*β*-nortropinol, 3*β*-phenyl-6*β*-acetoxynortropane (**10a**, *K*_*i*_ for M_2_ > 1 *μ*M), and 3*α*-hydroxy-3*β*-phenyl-6*β*-acetoxynortropane(**10b**, *K*_*i*_ for M_2_ > 1 *μ*M) to the binding of [^3^H]NMS to the muscarinic receptor subtypes M_1_–M_3_. In the curves of (a, c, e) (derivatives **5a**, **5b**, **5c**), data are expressed as means ± SEM from 3 samples in a range of 1.0 · 10^−10^ M to 1.0 · 10^−4^ M of competitor concentrations. In the curves of (b, d, f) (derivatives **5a**, **10a**, **10b**), data are expressed as means ± SEM from 4 samples in a range of 1.0 · 10^−10^ M to 1.0 · 10^−5^ M of competitor concentrations. Atropine curves are also displayed as a reference.

In Figure 2, the results of the competitive binding experiments are displayed. The affinity of 6*β*-acetoxynortropane, relative to [^3^H]NMS, for the muscarinic M_2_ receptor subtype proved to be high in both experiments. In the first experiment the *K*_*i*_ of 6*β*-acetoxynortropane was determined as 88.1 ± 23.8 nM (average ± SD; *n* = 3) and in the second experiment as 71.6 ± 4.8 nM (average ± SD; *n* = 4). In our experiments, selectivity ratios of the compound for the M_2_ over M_1_ or M_3_ receptor subtype proved to be approximately 65 and 70, respectively. 

The 6*β*-4′-iodobenzyl ether of 6*β*-nortropinol (**5b**) performed substantially less than 6*β*-acetoxynortropane and displayed a *K*_*i*_ of only 3.0 ± 0.7 *μ*M, while selectivity for the M_2_ receptor was lost. The selectivity ratios of this derivative for the M_2_ over the M_1_ and M_3_ receptors of the compound were determined as 0.1 and 0.2, respectively. 

The 6*β*-4′-iodobenzoate ester of 6*β*-nortropinol (**5c**) also performed less than 6*β*-acetoxynortropane, and a *K*_*i*_ of 6.8 ± 1.5 *μ*M was estimated for the M_2_ receptor, while selectivity ratios over the M_1_ and M_3_ receptor, were determined as 0.6 and 2.0, respectively.

The second series of experiments (Figure 2), using 3*β*-phenyl-6*β*-acetoxynortropane (**10a)** and 3*α*-hydroxy-3*β*-phenyl-6*β*-acetoxynortropane (**10b**) as competitors, likewise showed weak affinity for muscarinic receptors, and small competitive effects to the binding of [^3^H]NMS were only detected at the highest concentration of the tested range. The affinity for the muscarinic receptors could therefore not be assessed for these two derivatives.

## 4. Discussion

In the present study, we have synthesized derivatives that are based on 6*β*-acetoxynortropane, a tropane alkaloid described by Pei and coworkers, which was shown to be a muscarinic agonist with high affinity to muscarinic M_2_ receptor subtypes, but lower affinity to other muscarinic receptor subtypes [27]. Due to the apparent selectivity of 6*β*-acetoxynortropane for the M_2_ receptor, the compound may be of interest for use as a muscarinic receptor radiotracer. 

Two analogues of the tracer were synthesized in which the acetyl ester moiety on the 6*β*-position was replaced by either 4′-iodobenzyl ether (**5b**) or a 4′-iodobenzoate ester (**5c**). The competitive binding assays demonstrated that the substitution on the 6*β*-position of the tropane skeleton had shifted the affinity from the nanomolar range to the micromolar range and that the selectivity of the alkaloid for the M_2_ receptor subtype was lost. Therefore, two other analogues were synthesized retaining the 6*β*-acetoxy function, with substitution of a phenyl moiety on the 3*β*-position of the tropane skeleton: 3*β*-phenyl-6*β*-acetoxynortropane (**10a**) and 3*α*-hydroxy-3*β*-phenyl-6*β*-acetoxynortropane (**10b**). Unfortunately, these derivatives demonstrated even less favorable affinity for the three tested muscarinic receptor subtypes.

The challenge of the present study was to create a derivative of 6*β*-acetoxynortropane that is suitable for (radio)iodination, while preserving the affinity for the M_2_ receptor, optimizing lipophilicity to allow optimal blood-brain-barrier (BBB) penetration and to limit nonspecific uptake, maintaining the size of the molecule as small as possible, while not compromising metabolic stability.

In an earlier study, our group evaluated the potential M_2_ receptor tracer E-iodopentenyl-thio-TZTP, which showed moderate selectivity for the muscarinic M_2_ receptor over the M_1_ and M_3_ receptors in vitro [25], although selectivity for M_2_ receptors was less than the original FP-TZTP [10, 14]. However, in vivo experiments using the TZTP derivative proved to be unsuccessful due to high lipophilicity of the tracer and very rapid metabolism of the parent compound [10, 14]. The 6*β*-acetoxynortropane derivatives that were synthesized and evaluated in the present study have several advantages over the earlier tested TZTP derivative(s). The lipophilicity of derivative **5b** and **5c** or iodinated analogues of **10a** and **10b** is less than that of the earlier synthesized TZTPs, being within the estimated log *P* (*P* = partition coefficient in octanol-buffer at pH 7.4) range between 1 and 2 (data not shown), which is considered to be optimal for penetration of the BBB. Incorporation of an ester function such as in the 6*β*-4′-iodobenzoate ester of 6*β*-nortropinol (**5c**), 3*β*-phenyl-6*β*-acetoxynortropane (**10a**) or an additional hydroxyl group in 3*α*-hydroxy-3*β*-phenyl-6*β*-acetoxynortropane (**10b**) contributes to the reduction in lipophilicity as compared to the earlier reported TZTPs, which should theoretically limit nonspecific uptake of these potential tracers in the brain. Another advantage over the earlier tested TZTP derivatives is the position of the iodine atom. Although the previous TZTP derivatives also contained a sp2 carbon-bound iodine, this was substituted on the alkenyl side chain, whereas in the present 6*β*-4′-iodobenzyl ether (**5b**) and 6*β*-iodobenzoate ester of 6*β*-nortropinol (**5c**), the iodine is bound to an aromatic sp2 carbon atom, which favors the in vivo stability and prevents rapid deiodination. Although we did not test iodinated versions of 3*β*-phenyl-6*β*-acetoxynortropane (**10a**) and 3*α*-hydroxy-3*β*-phenyl-6*β*-acetoxynortropane (**10b**), the phenyl ring would also be the appropriate location for coupling of the iodine atom in these two compounds. Moreover, unlike the earlier described and apparently metabolically unstable TZTP derivatives [25], the 3*β*-phenyl-6*β*-acetoxynortropane (**10a**) and 3*α*-hydroxy-3*β*-phenyl-6*β*-acetoxynortropane (**10b**) should be more metabolically stable due to the direct substitution of the phenyl ring to the tropane skeleton, which is known to have favorable effects on the in vivo stability of tropane-derived radiotracers such as [^123^I]FP-CIT [31, 32] or [^123^I]*β*-CIT [33]. 

In our competitive binding experiments, the inhibition constant of the original compound 6*β*-acetoxynortropane (**5a**) was substantially higher than the *K*_*i*_ that was described by Pei and coworkers [27]. Reasons for this may include differences in the reference tracer, which was [^3^H]NMS in the present experiments, whereas Pei et al. used [^3^H]quinuclidinyl benzilate (QNB), as well as a difference between rat and human muscarinic receptors. Pei et al. demonstrated a *K*_*i*_ of 2.6 nM for 6*β*-acetoxynortropane at the muscarinic M_2_ receptor and very high selectivity ratios over either M_1_ or M_3_ receptors using the tritiated antagonist. In the same study, an even lower inhibition constant was reported by Pei et al. when using the muscarinic agonist [^3^H]oxotremorine as a reference. In our study, using only [^3^H]NMS but not [^3^H]oxotremorine as a reference, we calculated *K*_*i*_ values to the M_2_ receptor of 71 and 88 nM, in two separate series of competitive binding experiments using different protocols, and lower selectivity ratios to M_1_ and M_3_ receptors of 65 and 70, respectively. Nevertheless, such selectivity ratios would be very adequate for imaging of muscarinic M_2_ receptors in vivo. However, the iodinated analogues that were tested (**5b** and **5c)** only showed weak affinity for all three tested muscarinic receptor subtypes, whereas the *K*_*i*_ of either 3*β*-phenyl-6*β*-acetoxynortropane (**10a)** or 3*α*-hydroxy-3*β*-phenyl-6*β*-acetoxynortropane (**10b**) could not be assessed, but proved to be above the micromolar range. We tested the derivatives at a maximal concentration of 10^−5^ M, which may be a limitation of the present study, but *K*_*i*_ values in the micromolar range or higher were not considered of interest for our purposes. However, it cannot be excluded that iodination of derivative **10a** and **10b** would result in improved affinities for muscarinic receptors. Also, substitution of the phenyl group at the 3*α*-positition, which is known to have bulk tolerance in tropane-derived radiotracers (and muscarinic receptor antagonists such as atropine, NMS, and benztropine), may improve the in vitro binding characteristics.

In conclusion, we synthesized a series of analogues to 6*β*-acetoxynortropane, potentially being of interest for use as radiotracers for in vivo imaging of the muscarinic M_2_ receptor subtype in neurodegenerative or neuropsychiatric diseases. However, changing the original molecule on the 6*β*- or the 3*α*/*β*-position by substitution of a iodophenyl or phenyl ring severely reduced both the affinity and selectivity of the nortropane for the muscarinic M_2_ receptor subtype, and therefore, the synthesized analogues are not suitable for use in human SPECT imaging.
